# Do obstetric risk factors truly influence the etiopathogenesis of congenital muscular torticollis?

**DOI:** 10.1007/s10195-017-0461-z

**Published:** 2017-06-29

**Authors:** N. Hardgrib, O. Rahbek, B. Møller-Madsen, R. D. Maimburg

**Affiliations:** 1 Department of Pediatrics, Aarhus University Hospital, Aarhus N, 8200 Aarhus, Denmark; 2 Department of Children’s Orthopaedics, Aarhus University Hospital, Aarhus, Denmark; 3 Department of Obstetrics and Gynaecology, Aarhus University Hospital, Aarhus, Denmark

**Keywords:** Child, Congenital muscular torticollis, Obstetric, Perinatal, Risk factors

## Abstract

**Background:**

Congenital muscular torticollis (CMT) is seen in childhood and presents within months after birth. The etiology remains unknown; however, medical textbooks suggest trauma at birth as a main reason. The aim of this study was to systematically describe obstetric and perinatal outcomes in a population of children with a confirmed congenital muscular torticollis diagnosis.

**Materials and methods:**

Children with a validated diagnosis of congenital muscular torticollis born at Aarhus University Hospital from 2000 to 2014 were included in the study. Information on perinatal, intrapartum and neonatal characteristics were obtained from databases and from medical records, and systematically described.

**Results:**

In this study, there were no differences in birth characteristics in children with left- and right-sided torticollis, between boys and girls or between the conservatively treated and the children who needed surgery. Most of the children with congenital muscular torticollis in this study were delivered at term without signs of birth complications or trauma. None experienced moderate or severe asphyxia.

**Conclusions:**

The results of the present study suggests that complicated birth or birth trauma may not be the main cause of congenital muscular torticollis and point towards intrauterine and prenatal reasons for its development.

**Level of evidence according to OCEBM levels of evidence working group:**

3

## Introduction

Torticollis is a clinical diagnosis where the sternocleidomastoid muscle (SCM) is shortened on the involved side, leading to a lateral tilt towards the affected muscle and contralateral rotation of the face and chin [1–3]. Several obstetric and newborn risk factors have been proposed for the development of CMT, including prolonged labor, macrosomia, breech or other irregular fetal presentations [4–6]. The theory of birth trauma proposes disruption of the SCM muscle during the birth process [7], and medical text books state that trauma at birth is associated with CMT [8–10], although the true etiology remains unknown [7]. Recent research has proposed intrauterine risk factors [7, 11, 12], but only a few larger studies [6, 13, 14] have systematically collected information in an effort to describe the etiology, and none have used systematically collected obstetric outcomes.

The aim of this study was to describe obstetric outcomes in a population of children with a confirmed diagnosis of CMT

## Materials and methods

This study was designed as an observational case study of children referred to the Department of Children’s Orthopedics (DCO) at Aarhus University Hospital (AUH) between 1 January 2000 and 31 June 2014 with a diagnosis of CMT. AUH serves a population of ~325,000 inhabitants with 4500 deliveries annually. A tertiary neonatal intensive care unit (NICU) and orthopedic facilities for children are available at AUH. In Denmark, healthcare is financed by public taxes and includes antenatal, intrapartum and post-partum care.

The inclusion criteria were; children under the age of 18 years at time of referral and treatment for CMT, with a clinically confirmed CMT diagnosis, born at AUH. The diagnosis of CMT was based on the International Classification of Diseases, 10th revision (ICD-10), defined as torticollis ICD-10 codes DM436, DQ680A, DG243, and DP158A. Further, a post hoc examination of the medical record was made to ensure fulfillment of the diagnostic criteria in all included cases.

### Orthopedic data

We performed a retrospective examination of medical records from 2000 to 2014, using torticollis diagnosis codes. The medical records were reviewed by the first author (NH) to determine the specific diagnosis of CMT.

Cases were included if the symptoms were consistent with torticollis (lateral tilt and contralateral rotation of the face and chin, restricting movement) and stated in the medical record at the time of initial evaluation. Cases were excluded if history and physical examination were inadequate to confirm a diagnosis of torticollis. The following information was required for all included patients: gender, age at time of diagnosis, affected side of the torticollis, and history of prior treatment. Children with torticollis were classified as having CMT or non-CMT.

### Obstetric data

For children born at AUH, information about the birth process was retrieved from the Aarhus Birth Cohort (ABC). The ABC contains information on all deliveries at AUH. After delivery, the attending midwife enters information on the course of delivery and newborn status in a structured birth registration form into the birth cohort database. Information about the course of pregnancy and birth includes: parity (nullipara/multipara), in vitro fertilization (IVF) pregnancy, singleton pregnancy, gestational age, fetal presentation, augmention of labor (syntocinon^®^), induction of labor (prostaglandin, artificial rupture of membranes), colour of amnion fluid, delivery mode and duration of the second stage of labor. Information about the newborn includes: gender (male/female), Apgar score, umbilical cord pH, umbilical cord base excess (BSE), infant weight, infant length, infant head circumference, and transfer to the NICU.

## Results

**Fig. 1:**
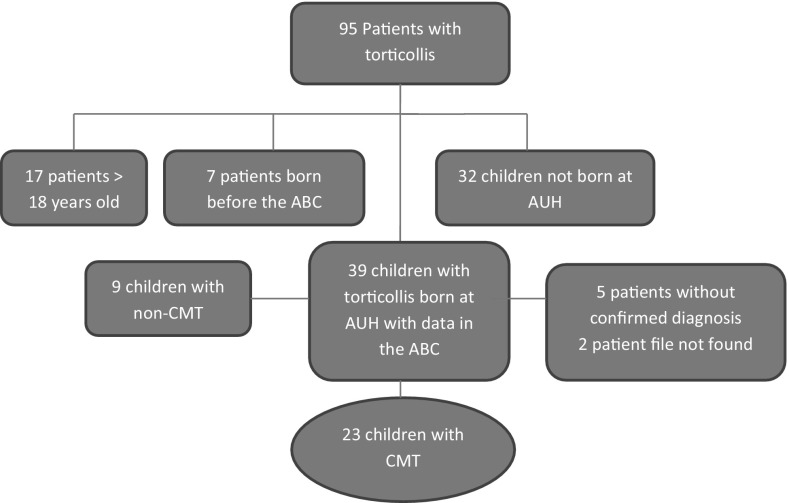
Flowchart of patient recruitment

In total 95 patients had been referred to DCO with torticollis in the study period. Of these, 17 patients were excluded because they were older than 18 years at the time of referral. Seven patients were born before the ABC was established and 32 patients were born outside AUH, leaving 39 children with torticollis fulfilling the inclusion criteria. Five had been admitted to the emergency department, but the diagnosis could not be confirmed, two patient’s medical records were missing, nine children had non-CMT and 23 children had CMT (Fig. 1). Of these, 13 had left-sided torticollis and 10 had right-sided. Fourteen children were treated conservatively and nine children had one operation or more.

Table 1 presents perinatal and obstetric outcomes and shows the following.

**Table 1:** Perinatal and obstetric outcome in 23 children with a diagnosis of torticollis born at Aarhus University Hospital from 2000 to 2014

Pregnancy	Gender	IVF pregnancy	Singleton	Parity	Gestational age	Fetal presentation	Augmention (Syntocinon^®^)	Induction	Amnion fluid	Delivery mode	Duration of stage 2 (min)
#1	Boy	No	Yes	Nulli	41 + 2	Vertex	Yes	No	Clear	Vaginal	38
#2	Girl	No	Yes	Nulli	39 + 5	Vertex	Yes	No	Thick green	Vaginal	62
#3	Boy	No	Yes	Nulli	40 + 1	Vertex	No	Prostaglandin	Clear	Vaginal	40
#4	Boy	No	Yes	Nulli	40 + 6	Vertex	No	No	Light green	Metal cup	4
#5	Girl	No	Yes	Nulli	40 + 0	Cephalic^a^	Yes	No	Clear	CS acute	–
#6	Boy	No	Yes	Nulli	40 + 2	Vertex	No	No	Green	Vaginal	54
#7	Girl	No	Yes	Nulli	40 + 2	Vertex	No	No	Clear	Vaginal	31
#8	Girl	No	Yes	Nulli	41 + 3	Vertex	Yes	No	Clear	Vaginal	19
#9	Girl	No	Yes	Nulli	40 + 0	Breech	No	Prostaglandin	Clear	Vaginal/assisted	16
#10	Boy	No	Yes	Multi	39 + 1	Breech	No	No	Clear	CS planned	–
#11	Boy	No	Yes	Nulli	41 + 0	Vertex	No	No	Clear	Vaginal	16
#12	Boy	No	Yes	Nulli	39 + 3	Vertex	No	No	Clear	Soft rubber cup	30
#13	Boy	No	Yes	Nulli	36 + 6	Vertex	No	No	Clear	Soft rubber cup	42
#14	Girl	No	No	Multi	37 + 3	Cephalic^a^	No	No	Clear	CS planned	–
#15	Boy	No	Yes	Nulli	40 + 0	Breech	Yes	Prostaglandin	Thick green	Vaginal/assisted	10
#16	Girl	No	Yes	Nulli	39 + 6	Vertex	Yes	Prostaglandin	Clear	Vaginal	19
#17	Girl	No	Yes	Nulli	42 + 0	Vertex	Yes	Prostaglandin	Light green	Metal cup	47
#18	Boy	Yes	No	Nulli	37 + 0	Vertex	Yes	No	Unknown	CS acute	–
#19	Boy	No	Yes	Nulli	34 + 4	Vertex	No	No	Clear	Vaginal	22
#20	Girl	No	Yes	Nulli	40 + 0	Vertex	No	No	Light green	Vaginal	10
#21	Boy	No	Yes	Multi	38 + 4	Vertex	No	No	Clear	Vaginal	13
#22	Boy	No	Yes	Missing	36 + 0	Breech	Yes	No	Clear	CS acute	–
#23	Girl	No	Yes	Nulli	39 + 6	Vertex	Yes	Ruptures of membranes	Clear	Metal cup	105

*CS* cesarean section

^a^Not specified

Of the children born at AUH, 19 were borne by nulliparous women and three by multiparous women. One was assigned with unknown parity. Two pregnancies were gemelli, and one of the twin pregnancies was conceived by IVF. Six women had their birth induced, five with prostaglandin and one with artificial rupture of the membranes, and a total of ten women received syntocinon to augment labor. In total, 17 children were in vertex presentation, two in unspecified cephalic presentation and four in breech presentation. Five children were delivered by cesarean section. Of those born vaginally, five children were delivered by vacuum extraction, and two children were assisted vaginal breech births. The second stage of labor for the vaginal births was on average 32 min, ranging from 4 to 105 min.

There were 10 girls and 13 boys. Most children were born at term between 37 + 0 weeks of gestation and 42 + 0 weeks of gestation. However, three were born near-term at 34 + 4, 36 + 0 and 36 + 6 weeks of gestation. All but two children had full Apgar score after 5 min and none of the children experienced moderate or severe asphyxia or acidosis (pH < 7.10 and BSE ≥ −10 mmol/l) during birth as measured in umbilicus cord blood. Four children were admitted to the neonatal ward; one due to thick meconium-stained amnion fluid and respiratory distress together with an affected Apgar score (6/1, 8/5), and two children after emergency cesarean section, due to asymmetric head shape and low birth weight, and the last child was referred for antibiotic treatment because of prolonged rupture of membranes.

The median birth weight was 3259 g, ranging from 2260 to 3990 g. The median length was 49.2 cm, ranging from 41 to 55 cm and the median head circumference was 34 cm for the 23 children (one child’s head circumference was not measured). Table 2 presents neonatal and treatment outcomes in the 23 children with a diagnosis of CMT born at AUH.

**Table 2:** Neonatal and treatment outcomes in 23 children with a diagnosis of torticollis born at Aarhus University Hospital from 2000 to 2014

Number	Apgar Scores 1 min/5 min	Umbilical cord pH	Umbilical cord BSE	Fetal weight (g)	Fetal length (cm)	Fetal head circumference (cm)	NICU	Side of torticollis	Treatment
#1	9/10	a 7.29	−5.7	3990	55	37	No	Left	Conservative
#2	6/8	v 7.19	−7.9	3280	52	33	Yes	Right	Operated × 3
#3	10/10	a 7.22	−3	3640	52	38	No	Right	Conservative
#4	8/9	a 7.12	−5	3680	54	37	No	Left	Conservative
#5	10/10	Missing	Missing	3910	53	35	No	Left	Operated × 3
#6	10/10	v 7.31	−2	3400	52	33	No	Left	Conservative
#7	10/10	a 7.28	−8	3270	53	35	No	Right	Conservative
#8	9/10	a 7.27	−5	3160	51	34	No	Left	Conservative
#9	6/10	a 7.40	−4.6	3350	55	36	No	Right	Operated × 1
#10	10/10	v 7.38	−1	3590	51	36	Yes	Right	Operated × 2
#11	10/10	v 7.30	Missing	3850	52	32	No	Left	Conservative
#12	10/10	v 7.43	−5	2620	48	34	No	Left	Conservative
#13	10/10	a7.27	−8	3240	50	35	No	Right	Operated × 1
#14	10/10	a 7.31	−1	2610	47	32	No	Left	Conservative
#15	9/10	v 7.36	Missing	2680	48	Missing	No	Left	Conservative
#16	10/10	a 7.35	−2	2700	49	32	No	Right	Operated × 2
#17	7/10	a 7.18	−6	3630	54	34	No	Right	Conservative
#18	10/10	Missing	Missing	3515	51	36	No	Right	Conservative
#19	10/10	v 7.21	−3	2260	54	34	No	Left	Conservative
#20	9/10	v 7.19	−5.5	3560	50	34	No	Left	Operated × 1
#21	10/10	7.41	−1	2850	41	35	No	Left	Conservative
#22	8/10	7.11	Missing	2330	49	33	Yes	Left	Operated × 3
#23	5/10	v 7.22	−10	3830	51	36	Yes	Right	Operated × 1

*a* arterial, *v* venous, *NICU* neonatal intensive care unit

## Discussion

In this observational case study we systematically reviewed the obstetric outcomes in a population of children with a confirmed diagnosis of CMT and found that there were no differences in birth characteristics in children with left- and right-sided CMT, between boys and girls or between the conservatively treated and the children who needed surgery. The children were primarily born by nulliparous women. Most were delivered without any trauma, seven experienced an assisted delivery, either by vacuum extraction or assisted breech birth, and with a mean second stage of labor of 32 min. Complicated birth, as measured by Apgar score, umbilical cord pH, and umbilical cord base excess, indicated that none of the children suffered moderate or severe asphyxia. Three initially had low Apgar score but all children had normal scores after 10 min. Most of the children with CMT in this study were delivered at term without signs of birth complications or trauma and none of the children could be classified as macrosomia.

Comparing our data with the existing literature we found two studies suggesting that the side of the torticollis is related to CMT either by intrauterine positioning [15] (head positioning in utero can selectively injure the SCM muscle) or due to delivering of the first shoulder [16]. Information about first delivered shoulder, the final fetal position being either left occipital or right occipital, was not available in the ABC cohort. Moreover, ultrasound is only done as routine in Denmark at around gestational weeks 12 and 19 and could therefore not provide further information about the specific fetal position during pregnancy.

No previous studies with information about parity, augmentation, or induction of labor are available. A case–control study [7] examined gestational age and birth weight for CMT patients, but not in relation to complicated birth or developing CMT. Several have studied fetal presentation and delivery mode related to CMT [7, 11, 12, 15–19]. To our knowledge no former studies have examined the duration of second stage of labor, Apgar score, umbilical cord pH and base excess, infant head circumference and transfer to the neonatal ward as indicators of complicated birth.

In our data, we found a lower prevalence of breech presentation in children with CMT, compared to earlier studies [17]. Half of the children in breech presentation were delivered vaginally and the other half by cesarean section. In a former case control study with 178 patients, Lee et al. [7] compared vaginal births with cesarean sections and found no difference in the clinical severity of CMT according to the mode of delivery, suggesting that prenatal factors most likely cause CMT due to the reduced risk of birth trauma in cesarean sections. This is in accordance with two case reports [12, 15], questioning the traumatic vaginal breech delivery theory as being the dominant pathophysiology behind CMT.

Other studies questioned trauma and difficult birth, and instead pointed towards sequelae from intrauterine and prenatal factors as the main cause of CMT. Stellwagen et al. [11] found an association between torticollis and the fetus being in the same intrauterine position for more than 6 weeks before delivery and Davids et al. [16] used magnetic resonance imaging (MRI) to observe the SCM muscle in infants and found signals similar to those in compartment syndrome.

In contrast, Hollier et al. [19] found a high frequency of complications during pregnancy and delivery in their small retrospective study of 11 patients, and Ho et al. [18] found higher rates of assisted breech births, instrumental deliveries and cesarean sections, which led them to conclude that birth trauma appears to be the main etiological factor in CMT. Suzuki et al. [17] suggested that stretching of the SCM muscle during delivery may be a direct cause of CMT. In our population only a few cases experienced moderate birth trauma: mainly those delivered with vacuum extraction. In general, most of the studies [17–19] had only examined fetal presentation and delivery mode, lacking more specific information from obstetric and neonatal medical records.

There seems to be a tendency towards intrauterine and prenatal cause, but the possibility of a perinatal trauma to the SCM muscles cannot be excluded. In our study, most of the children with CMT were born after uncomplicated deliveries, contradictory to the most common theories described in medical textbooks.

However, our study has some limitations. Primarily it only included 23 cases of CMT. One reason for this is that 90–95% of CMT resolves within a year by manual stretching and therefore the majority of these children are never referred to an orthopedic facility. It is therefore expected that children included in this study represent the more severe cases. Our study size was further limited by including only children born at AUH, as this was the only hospital where we were able to retrieve validated obstetric data. However, we believe the study sample to be representative.

We were unable to retrieve family history of CMT in the patient records. A potential genetic association may accumulate cases of CMT within families.

Finally, this study was a retrospective observational case study with prospective collected obstetric information. Retrospective studies are useful for studying diseases with low incidence. A large prospective cohort study with evaluation of fetal positioning during pregnancy with systematic examination of the SCM in both the perinatal and the neonatal period using ultrasound or MRI, together with collection of obstetric information may provide further information of CMT etiology.

The results of the present study contribute to existing knowledge by pointing mainly towards intrauterine and prenatal reasons for developing CMT, and indicate that complicated birth and trauma may not be the main cause of CMT, even though this is stated in pediatric orthopedic textbooks.
